# New Insights into the Melanophilin (*MLPH*) Gene Affecting Coat Color Dilution in Rabbits

**DOI:** 10.3390/genes9090430

**Published:** 2018-08-23

**Authors:** Julie Demars, Nathalie Iannuccelli, Valerio Joe Utzeri, Gerard Auvinet, Juliette Riquet, Luca Fontanesi, Daniel Allain

**Affiliations:** 1 GenPhySE, INRA Animal Genetics, Toulouse Veterinary School (ENVT), Université de Toulouse, 31326 Castanet Tolosan, France; nathalie.iannuccelli@inra.fr (N.I.); juliette.riquet@inra.fr (J.R.); daniel.allain@inra.fr (D.A.); 2 Department of Agricultural and Food Sciences (DISTAL), Division of Animal Sciences, University of Bologna, 40127 Bologna, Italy; valeriojoe.utzeri2@unibo.it (V.J.U.); luca.fontanesi@unibo.it (L.F.); 3 GenESI, INRA le Magneraud, 17700 Surgères, France; gerard.auvinet@inra.fr

**Keywords:** coat color dilution, melanophilin, rabbit

## Abstract

Coat color dilution corresponds to a specific pigmentation phenotype that leads to a dilution of wild type pigments. It affects both eumelanin and pheomelanin containing melanosomes. The mode of inheritance of the dilution phenotype is autosomal recessive. Candidate gene approaches focused on the melanophilin (*MLPH*) gene highlighted two variants associated with the dilution phenotype in rabbits: The c.111-5C>A variant that is located in an acceptor splice site or the c.585delG variant, a frameshift mutation. On the transcript level, the skipping of two exons has been reported as the molecular mechanism responsible for the coat color dilution. To clarify, which of the two variants represents the causal variant, (i) we analyzed their allelic segregation by genotyping Castor and Chinchilla populations, and (ii) we evaluated their functional effects on the stability of *MLPH* transcripts in skin samples of animals with diluted or wild type coat color. Firstly, we showed that the c.585delG variant showed perfect association with the dilution phenotype in contrast to the intronic c.111-5C>A variant. Secondly, we identified three different *MLPH* isoforms including the wild type isoform, the exon-skipping isoform and a retained intron isoform. Thirdly, we observed a drastic and significant decrease of *MLPH* transcript levels in rabbits with a coat color dilution (*p*-values ranging from 10^−03^ to 10^−06^). Together, our results bring new insights into the coat color dilution trait.

## 1. Introduction

Different coat colors in the European rabbit (*Oryctolagus cuniculus*) have been selected throughout domestication and are nowadays fixed in specific breeds. Among the various phenotypic traits, coat color dilution corresponds to an altered distribution of eumelanin and pheomelanin pigments in skin and hair [1]. A similar coat color dilution phenotype has been observed in other mammals such as mice. Mutations within proteins encoding the melanosome transport complex were described in the myosin VA (*Myo5a*) [2], Ras-related protein (*Rab27a*) [3] and melanophilin (*Mlph*) [4] genes. In humans, patients suffering from Griscelli Syndrome (GS) exhibit a pigment dilution in their hair and skin, which, depending on the specific genetic variant, may or may not be accompanied by other important symptoms. Interestingly, mutations within *MYO5A* [5], *RAB27A* [6] and *MLPH* [7,8] are respectively responsible for GS1 (OMIM #214450), GS2 (OMIM #607624) and GS3 (OMIM #609227). Only GS3 is characterized by hypomelanosis with no immunologic or neurologic manifestations.

Candidate gene approaches focused on these specific genes were performed in several species presenting a dilution-like coat color phenotype. The mode of inheritance of the dilution phenotype is autosomal recessive [1]. Mutations within the *MLPH* gene were identified in mice [4], cat [9] (OMIA 000206-9685), dog [10,11,12] (OMIA 000031-9615), chicken [13] (OMIA 001445-9031), quail [14] (OMIA 001445-93934), American mink [15] (OMIA 001438-452646) and cattle [16] (OMIA 001438-452646). In rabbits, although variants have also been highlighted within the *MLPH* gene, two published studies have suggested different variants as the genuine causal mutation [17,18]. An alternatively spliced *MLPH* transcript isoform corresponding to two-exon skipping was suggested as the causal molecular mechanism for the coat color phenotype [17]. The c.111-5C>A variant, located within intron 2 in an acceptor site for splicing, was reported as the most likely variant leading to this exon skipping [17]. This skipping of exons 3 and 4 caused a frameshift leading to a change of two amino acids followed by a premature stop codon [17]. A second variant, c.585delG, corresponding to a 1-bp deletion in exon 6 of the *MLPH* gene has also been identified in various breeds with a dilution of their coat [17,18]. This variant also led to a frameshift and an altered amino acid sequence with a premature stop codon. Both variants were identified in the first study and are associated with coat color dilution in several breeds (Netherland Dwarf, Loh, Lionhead Dwarf and Blue Vienna) [17]. The authors suggested a higher relevance of the c.111-5C>A variant although an effect of the c.585delG variant on the dilution phenotype was suggested for individuals who were not homozygous for mutated allele at c.111-5C>A [17]. However, only the c.585delG variant was highlighted and analyzed by Fontanesi et al. [18]. An association signal with the coat color dilution was obtained in various breeds (*n* = 7) including Blue Vienna and Castor Rex [18].

To better understand the dilution phenotype in rabbits and to likely discriminate, which of the two previously reported variants is the true causal variant, we analyzed their segregation with the dilution phenotype in Castor and Chinchilla breeds already known to have the trait of interest [18]. Once highlighting the genetic implication of the c.585delG variant, we evaluated its impact on *MLPH* transcripts in order to understand the molecular mechanisms involved in coat color dilution in rabbits.

## 2. Materials and Methods

### 2.1. Animals

**Figure 1:**
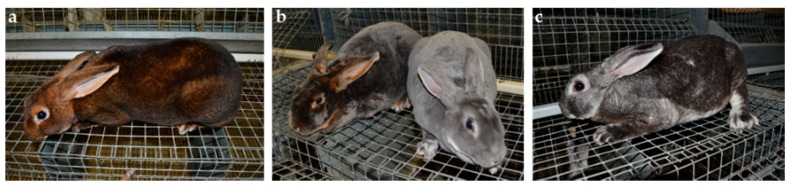
Characterization of the dilution phenotype in Castor and Chinchilla breeds. The rabbits with a wild type (wt) coat color are shown for the (**a**) Castor and (**c**) Chinchilla breeds. The picture (**b**) represent Castor (left) and Chinchilla (right) diluted animals.

The coat color dilution phenotype was observed in Castor and Chinchilla breeds (Figure 1). Both breeds are selected for fur production; each includes two lines. The wild type (wt) Castor line has a normal black-brown coat color with a yellow agouti band. The diluted Castor line was derived from the wt Castor line by selecting the blue coat color phenotype. Similarly, the wt Chinchilla line carries a normal black-brown coat color with the Ch allele at the C locus and the other line (diluted Chinchilla) was derived from wt Chinchilla with the ash coat color phenotype. Nowadays, the four lines represent four distinct populations since they are selected independently.

A first set of 74 rabbits including 50 Castor (37 wt vs. 13 diluted) and 24 Chinchilla (12 wt vs. 12 diluted) was used for genotyping on the genomic DNA (gDNA) and complementary DNA (cDNA) level. Moreover, 5 animals out of 74 were entirely sequenced for the *MLPH* gene. Among the first set, 43 rabbits were selected based on their phenotype and their genotype at the c.585delG variant to investigate *MLPH* transcripts. Within this selection, 17 individuals were diluted and homozygous mut/mut (8 Castor and 9 Chinchilla); 8 Castor wt animals were heterozygous wt/mut, and 18 rabbits were wt and homozygous wt/wt (8 Castor and 9 Chinchilla). Unrelated individuals were chosen.

Table S1 integrates the full information concerning animals used in this article.

### 2.2. Samples

All procedures were conducted in accordance with the Guide for the Care and Use of Agricultural Animals in Research and Teaching.

Skin samples were collected in 2012; no ethical code was needed at that time in France. Skin samples were collected at 3 months of age for all rabbits. Skin punch biopsies (28 mm^2^) were taken from the breast by an expert technician. Genomic DNA and total RNA were extracted from the same biopsy using the AllPrep DNA/RNA Mini kit according to the manufacturer’s protocol (Qiagen, Venlo, Netherlands). RNA samples were DNAse-treated to avoid gDNA contamination and diluted at 50 ng/µL in RNAse-free water. RNA (1 µg) was reverse-transcribed using Superscript II reverse transcriptase (Invitrogen, Cergy-Pontoise, France) and random primers (Nonamers, Sigma-Aldrich, St. Louis, MO, USA). Synthetised cDNA was used for real-time quantitative PCR.

### 2.3. Amplification of MLPH gDNA and cDNA for Sequencing

Nineteen PCR primers pairs [18] were used for sequencing *MLPH* gDNA. Briefly, PCR was carried out using a 2720 thermal cycler (Life Technologies, Carlsbad, CA, USA) in a 22-µL reaction volume containing 20 ng gDNA, 0.5 U GoTaq DNA polymerase (Promega, Madison, WI, USA), 1X GoTaq PCR buffer, 0.2 mm dNTPs, 0.4 µm of each primer and 1.5 mm MgCl_2_. The PCR profile was as follows: 5 min at 95 °C, 35 amplification cycles of 30 s at 95 °C, 30 s at 62 °C or 64 °C, 30 s at 72 °C, 10 min at 72 °C.

Five additional primers pairs were used (Table S2) and were designed on the rabbit *MLPH* gene sequence (ENSOCUG00000016496) with Primer3plus software (http://www.bioinformatics.nl/cgi-bin/primer3plus/primer3plusAbout.cgi). Two Long Range PCRs were carried out using a 2720 thermal cycler (Life Technologies) in a 23-µL reaction volume containing 100 ng gDNA, 1X GoTaq Long PCR Master Mix (Promega), 0.2 mm dNTPs, 0.4 µm of each primer and 1.5 mm MgCl_2_. The PCR profile was as follows: 2 min at 94 °C, 35 amplification cycles of 20 s at 94 °C, 5 min at 68 °C, 10 min at 72 °C. Three other pairs were used for sequencing *MLPH* cDNA. PCR was carried out using a 2720 thermal cycler (Life Technologies) with the GC-Rich PCR System (Roche, Basel, Switzerland) in a 50-µL reaction volume containing 5 µL cDNA dilution in 1/5, 2 U GC-rich enzyme, 1X buffer, 0.2 mm dNTPs, 0.4 µm of each primer and 1.5 mm MgCl_2_. The PCR profile was as follows: 5 min at 95 °C, 45 amplification cycles of 30 s at 95 °C, 1 min at 60 °C or 62 °C, 30 s at 72 °C, 10 min at 72 °C.

Sequencing of PCR products was performed using an ABI Big Dye terminator v3.1 sequencing kit (Life Technologies) as recommended by the manufacturer’s protocol. Briefly, excess of PCR primers was removed via the treatment of PCR products with 10 U Exonuclease (New England Biolabs, Ipswich, MA, USA) and 0.5 U Thermo Sensitive Alkaline Phosphatase (Promega) for 45 min at 37 °C and 30 min at 80 °C. Purified PCR amplicons were then sequenced using the ABI Big Dye terminator v3.1 sequencing kit (Life Technologies,) and purified again with the Sephadex™ G-50 Superfine (GE Healthcare, Chicago, IL, USA) before being analysed on an automated ABI 3730 capillary sequencer (Life Technologies). Electropherograms were analysed with ChromasPro software (Technelysium, South Brisbane, Australia).

### 2.4. Genotyping Analyses

#### 2.4.1. Genotyping of the c.585delG Variant on gDNA

Allele specific PCR was used for genotyping the c.585delG variant. Primers used (Table S2) were designed on the rabbit *MLPH* gene sequence (ENSOCUG00000016496) with Primer3plus software (http://www.bioinformatics.nl/cgi-bin/primer3plus/primer3plusAbout.cgi). PCR was carried out using a 2720 thermal cycler (Life Technologies) in a 12-µL reaction volume containing 20 ng gDNA, 0.5 U GoTaq DNA polymerase (Promega), 1X GoTaq PCR buffer, 0.2 mm dNTPs, 0.4 µm of each primer and 1.5 mm MgCl_2_. The PCR profile was as follows: 5 min at 95 °C, 35 amplification cycles of 30 s at 95 °C, 30 s at 66 °C for the mutated allele or 68 °C for the wild type allele, 30 s at 72 °C, 10 min at 72 °C. PCR products were electrophoresed on 2% agarose gel and visualized with bromide of ethidium.

#### 2.4.2. Genotyping of the c.111-5C>A Variant on gDNA

Sequencing was used for genotyping the c.111-5C>A variant. Primers used (Table S2) were designed on the rabbit *MLPH* gene sequence (ENSOCUG00000016496) with the Primer3plus software (http://www.bioinformatics.nl/cgi-bin/primer3plus/primer3plusAbout.cgi). PCR and sequencing of PCR products were performed as previously mentioned.

#### 2.4.3. Genotyping of the c.585delG Variant on cDNA

The c.585delG variant was analyzed on cDNA using Restriction Fragment Length Polymorphism (RFLP). The primers used (Table S2) were designed on the rabbit *MLPH* gene sequence (ENSOCUG00000016496) with Primer3plus software (http://www.bioinformatics.nl/cgi-bin/primer3plus/primer3plusAbout.cgi). PCR was carried out using a 2720 thermal cycler (Life Technologies) with the GC-Rich PCR System (Roche) in a 25-µL reaction volume containing 2.5 µL cDNA dilution in 1/5, 1 U GC-rich enzyme, 1X buffer, 0.2 mm dNTPs, 0.4 µm of each primer and 1.5 mm MgCl_2_. The PCR profile was as follows: 5 min at 95 °C, 45 amplification cycles of 30 s at 95 °C, 30 s at 56 °C, 30 s at 72 °C, 10 min at 72 °C. Digestion was performed with SmlI enzyme (New England Biolabs) in a 12-µL reaction volume containing 4 µL PCR product, 1 U enzyme, 1X buffer at 55 °C overnight. The digestion products were electrophoresed on 2% agarose gel and were visualized with bromide of ethidium. In animals that were homozygous wt/wt for the c.585delG variant, a 173-bp undigested fragment was expected, while in rabbits homozygous mut/mut, digestion by SmlI was suggested, leading to two fragments of sizes 123 and 50 bp.

### 2.5. Stability and Accumulation of MLPH Transcripts

Primers are listed in Table S2. Real-time quantitative PCR analysis was carried out with a LightCycler^®^ 480 System using the SYBR Green I Master (Roche Life Science, Indianapolis, IN, USA) as recommended by the manufacturer. Briefly, 3 µL of diluted cDNA (1/5) were mixed with 0.15 µM of each primer and 1X Master volume in a final volume of 10 µL, and a standard qPCR amplification was then performed as follows: 45 cycles of 15 s at 95 °C, 15 s at 60 °C and 15 s at 72 °C. Each qPCR reaction was done in duplicate.

A final melting curve analysis was systematically performed for each primer pair to check for the presence of only one PCR product peak, and each amplicon was sequenced to verify the amplification specificity (as explained previously). The baseline adjustment method of the LightCycler 480 software (Roche Diagnostic, Rotkreuz, Switzerland) was used to determine the Ct (Cycle threshold) in each reaction. Primer efficiency curves were generated using five serial dilutions of cDNA (two-fold dilution from 1:5 to 1:80) on the abscissa and the corresponding Ct on the ordinate. The slope of the log-linear phase reflects the amplification efficiency (E) derived from the formula E = e(−1/slope). For quantification analysis, the Ct of target gene was compared with the mean of the expression of the internal reference gene *HPRT* encoding a ubiquitous hypoxanthine guanine phosphoribosyl transferase according to the ratio R = 100 × (E^ReferenceCt^ Reference/E^TargetCt^ Target). Results were expressed as the quantify relative to the mean of the housekeeping gene expression. The significance of results was tested using a *t*-test for the comparison of the genotypic clades.

## 3. Results

### 3.1. The Frameshift Mutation (c.585delG) Appeared to be the Putative Causal Variant

To discriminate between both variants for determining the putative causal variant for the coat color dilution in rabbits, we genotyped a total of 74 individuals from the Castor (37 wt vs. 13 diluted) and Chinchilla (12 wt vs. 12 diluted) breeds (Table S1). All diluted animals were homozygous for both mutatant alleles, not allowing the differentiation between both variants (Table 1) and suggesting total linkage disequilibrium. However, the linkage disequilibrium was broken in some of the wild type rabbits since 19 out of 49 carried two mutant alleles for the c.111-5C>A variant while being homozygous wt/wt or heterozygous wt/mut for the c.585delG variant (Table 1). These results suggested that only the 1-bp deletion corresponding to the mutant allele of the c.585delG variant was perfectly associated with the dilution phenotype in Castor and Chinchilla breeds.

**Figure 2:**
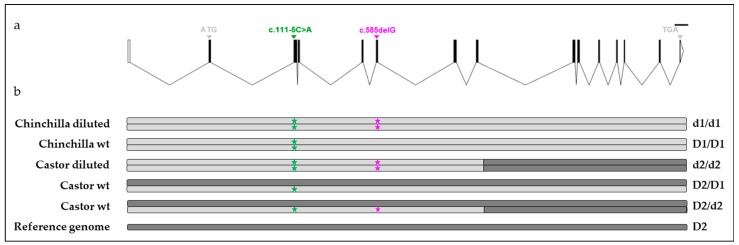
Characterization of haplotypes identified in Castor and Chinchilla breeds. (**a**) Graphical representation of the rabbit *MLPH* gene with the location of both the c.111-5C>A and c.585delG variants. The coding sequence is in black, with START and STOP codons in exons 2 and 15, respectively. (**b**) Graphical representation of identified haplotypes. A total of 118 variants including 31 exonic variants were phased to reconstruct haplotypes from five individuals (Tables S1 and S3). Two diluted (d1 and d2) and two wt haplotypes (D1 and D2) were identified. Green and pink stars represent mutant alleles for the c.111-5C>A and c.585delG variants, respectively.

We sequenced, from both gDNA and cDNA, the *MLPH* gene including all exons with 5′-UTR and 3′-UTR regions and exon-intron junctions in two Chinchilla (1 wt vs. 1 diluted) and three Castor (2 wt vs. 1 diluted) rabbits (Table S1). The whole coding sequence was obtained from exons 1 to 15 encoding a wt protein of 562 amino acids (Figure 2a, Figures S1 and S2). Among 118 identified variants, 31 were exonic, including 12 non-synonymous variants (Table S3). From the five sequenced animals, 1 diluted (haplotype d1) and 1 wild type (haplotype D1) haplotype were reconstructed in Chinchilla, and 1 diluted (haplotype d2) and 1 wild type (haplotype D2) haplotype were determined in Castor (Figure 2b and Table S3). The haplotype D2 is similar to the reference Californian sequence and the haplotype d2 corresponded to a recombinant haplotype between the diluted haplotype d1 and the wild type haplotype D2 (Figure 2b and Table S3). Importantly, only the 1-bp deletion at the c.585delG variant discriminated the diluted haplotype d1 from the wild type haplotype D1 in Chinchilla while both individuals were homozygous mut/mut at the c.111-5C>A variant (Figure 2b and Table S3). These data reinforced the 1-bp deletion (c.585delG) as the putative causal variant.

### 3.2. Three Different MLPH Transcripts Were Identified in Wt and Diluted Animals

**Figure 3:**
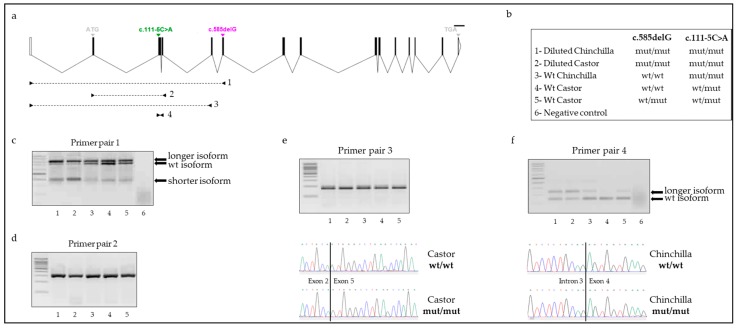
Identification of various *MLPH* isoforms. (**a**) Graphical representation of the rabbit *MLPH* gene with location of the c.111-5C>A and c.585delG variants. Locations of primer pairs used for RT-PCR are shown. (**b**) Five rabbits (3 Castor and 2 Chinchilla) were analyzed, and their phenotypes and genotypes at both variants are shown. For primer pairs 1 and 4, a negative control without reverse transcriptase was added since amplification might correspond to gDNA contamination. For all agarose gels, the order corresponds to numbers of animals. (**c**) Agarose gel using primer pair 1. (**d**) Agarose gel using primer pair 2. Primer pair 2 is specific to the wt *MLPH* isoform. (**e**) Results using primer pair 3. This primer pair is specific to the shorter *MLPH* isoform which is the *MLPH* transcript lacking exons 3 and 4 as shown on Sanger electropherograms. (**f**) Results using primer pair 4. PCR products were sequenced identifying a longer *MLPH* isoform containing intron 3 as shown on electropherograms.

To determine whether exon skipping caused by the c.111-5C>A variant might affect *MLPH* transcript production, we first identified *MLPH* transcripts. We amplified cDNA from the five previously sequenced animals using various pairs of primers (Table S2, Figure 3a,b and Figure S1). We identified three different *MLPH* transcript isoforms using a pair of primers amplifying exon 1 to exon 6 (Figure 3a–c): the expected wild type isoform, a shorter transcript corresponding to the expected size for skipping of exons 3 and 4 and a longer isoform. The various isoforms were present in the five individuals but the quantity appeared variable. Diluted animals that were homozygous mut/mut at the c.585delG variant showed less amplification of the wt *MLPH* isoform and more amplification of the shorter *MLPH* isoform compared to wt rabbits for the coat color dilution (Figure 3c).

We then designed pairs of primers that were specific to each *MLPH* isoform (Figure 3a and Figure S1). The wt *MLPH* isoform was detected in all individuals, but the amplification appeared similar between wt and diluted rabbits (Figure 3b,d). The shorter *MLPH* transcript was also amplified in all 5 animals (Figure 3b,e). The sequencing of the PCR product in two Chinchilla rabbits (1 wt vs. 1 diluted) confirmed that this isoform corresponded to an isoform without exons 3 and 4 (Figure 3e). Here, both wt and diluted animals showed the same amplification profile (Figure 3e) but none of the rabbits was wt/wt for c.111-5C>A, which did not allow us to conclude about the causal effect of this variant for the two-exon skipping.

The longer *MLPH* transcript was also identified using another pair of primers (Figure 3a,f). Animals with a dilute phenotype and homozygous mut/mut at both variants showed a stronger amplification of the longer isoform compared to wt individuals (Figure 3b,f). We sequenced this novel *MLPH* isoform and found a retained intron 3 transcript in both wt and diluted rabbits (Figure 3f). This PCR product likely corresponds to a novel *MLPH* isoform rather than a gDNA contamination given the result of the negative control.

### 3.3. MLPH Transcripts Were Significantly and Drastically Decreased in Diluted Animals

**Figure 4:**
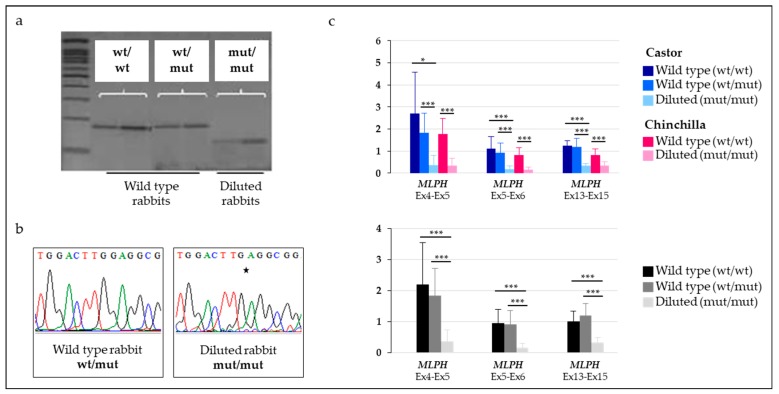
Accumulation and stability of *MLPH* transcripts in skin samples of rabbits with either wild type or dilution phenotype. (**a**) Semiquantitative analysis of *MLPH* transcripts by cDNA Restriction Fragment Length Polymorphism (RFLP) for the c.585delG variant in wild type and diluted rabbits. In wild type animals which are heterozygous, the deleted *MLPH* transcript was not observed. (**b**) Sequencing of cDNA. Two rabbits (1 wt vs. 1 diluted) that are, respectively, heterozygous and homozygous for the mutant allele at the c.585delG variant were sequenced. In the heterozygous animal, only the wild type *MLPH* transcript without the deletion was detected. (**c**) Quantification of the *MLPH* expression. At the top, Castor animals are in blue (dark, medium and light, respectively, for wt/wt, wt/mut and mut/mut at the c.585delG variant position) and Chinchilla rabbits are in pink (dark and light, respectively, for wt/wt and mut/mut at the c.585delG variant position). At the bottom, previous results were combined to compare animals given their genotype at the c.585delG variant position. Stars correspond to the significance from the performed *t*-test, where * is *p* < 0.05 and *** is *p* < 0.001.

To evaluate the putative functional effect of the c.585delG on the stability and accumulation of *MLPH* transcripts, we analyzed cDNA for the c.585delG variant. A dataset of 43 Castor and Chinchilla including wild type and diluted rabbits and the three groups of genotypes (wt/wt vs. wt/mut vs. mut/mut) at the c.585delG variant was used (Table S1). The *MLPH* transcript carrying the 1-bp deletion was detected by RFLP and sequencing but only in homozygous diluted animals (Figure 4a,b). In wt/mut heterozygous individuals, on the cDNA level only the wt allele could be detected (Figure 4a,b), suggesting a degradation of the mutant *MLPH* transcript with the 1-bp deletion. In addition, these data showed that the quantity of the mutated *MLPH* transcript seemed very weak compared to the wt transcript. To confirm this observation, we then measured the stability of *MLPH* transcripts in the skin of 43 rabbits from the two breeds selected on both their dilution phenotype and their genotype at the c.585delG variant (Table S1). We observed that *MLPH* transcripts were significantly reduced in diluted individuals which were homozygous for the deleted allele at the c.585delG variant while both heterozygous and homozygous wt/wt animals showed a similar profile (Figure 4c). These results suggested that the overall amount of *MLPH* transcripts was decreased in diluted rabbits without distinction of the various *MLPH* isoforms.

## 4. Discussion

Dilution of coat color is a trait of interest in many species. Variants within the melanophilin (*MLPH*) gene have been identified in dogs [10,11,12], mink [15] and cattle [16]. In humans, *MLPH* variants are responsible for GS3 (OMIM #609227) [7,8]. In rabbits, two different variants were suggested as potentially causal for the dilution phenotype. The first described variant [17], c.111-5C>A, was located within the second intron and resulted in a two-exon skipping *MLPH* transcript. This skipping of exons 3 and 4 caused a frameshift leading to a change of two amino acids followed by a premature stop codon at the fortieth amino acid of the protein [17]. The second variant [17,18], c.585delG, corresponded to a 1-bp deletion within the sixth exon of the *MLPH* gene, leading to a premature STOP codon at the 360th amino acid of the protein. To discriminate between the two variants and evaluate the effect on the stability and accumulation of *MLPH* transcripts, we performed both genetic and functional studies focused on *MLPH* in rabbits from the Castor and Chinchilla breeds in which the dilution phenotype segregated.

We managed to sequence the whole *MLPH* sequence from exons 1 to 15. This wt *MLPH* sequence obtained from both Castor and Chinchilla animals was similar to NM_001297485.1 [17] obtained from a black Loh rabbit. The rabbit MLPH protein seemed shorter with 562 amino acids (NP_001284414) compared to both human and mouse proteins consisting of 600 (NP_077006) and 590 (NP_443748) amino acids, respectively. While the beginning (amino acids 1 to 210) and the end (amino acids 460 to 560) of the protein are homologous to human and mice proteins, the middle of the protein is quite different in rabbit. Indeed, the Myosin VA binding domain appeared very different across species compared to Rab27A and Actin binding domains that seemed very well conserved among rabbit, human and mice.

The genetic analysis performed in Castor and Chinchilla breeds likely excluded the c.111-5C>A variant [17] as the causal mutation of the dilution phenotype. Indeed, some animals which were homozygous mut/mut at this variant and homozygous wt/wt or heterozygous at the c.585delG variant had a wt phenotype which is incompatible with the postulated causal role of this variant in an autosomal recessive mode of inheritance with full penetrance of the coat color dilution [1]. Moreover, only the c.585delG variant perfectly discriminated wt (D1) from diluted (d1) haplotypes in the Chinchilla breed as observed in [18] between the most frequent wt haplotype and diluted haplotype obtained from the *MLPH* sequencing of 18 rabbits of different coat color. While a few Castor individuals were analyzed by Fontanesi et al. [18], neither Castor nor Chinchilla rabbits were genotyped by Lehner et al. [17]. Different variants within the *MLPH* gene associated with the dilution phenotype would be one hypothesis explaining the discrepancy between our results and those of [17]. This was observed in sheep for various *BMP15* mutations that were all responsible for sterility in homozygous ewes [19,20,21,22,23] or in cattle for mutations in the myostatin gene (*MSTN*), which were all associated with hypermuscularity [24]. However, all diluted haplotypes identified from six different rabbits including one Chinchilla, one Castor, one Vienna Blue [18], one Checkered Giant [18] and two F1 Checkered Giant [18] were similar for all common positions. These results strongly suggested that in the investigated rabbit breeds a single *MLPH* variant influences coat color dilution instead of different variants within the *MLPH* gene, each specific to a breed. The 1-bp deletion in exon 6 of the *MLPH* gene might be the causal variant for the dilution phenotype in Castor and Chinchilla breeds. The same variant in both populations suggested a common origin with likely an introgression of Chinchilla into Castor given the shorter size of the Castor haplotype carrying the deletion. To go into further details about the origin of the rabbit dilute allele, it would be interesting to determine the shortest diluted haplotype segregating in breeds of rabbits with a dilution of their coat color.

Fancy breeders suggest the existence of two types of dilution: one that gives a darker grey (or blue), as observed in 2 rabbits [17], and another one that gives a lighter grey (or blue) colour. While both variants (c.111-5C>A and c.585delG variant) were identified in [17], authors suggested the c.111-5C>A as the most probable candidate due to the *p*-value of the trend test and the early position in the protein of a premature stop codon although the c.585delG variant showed the best association signal from both genotypic and allelic tests [17]. However, none of the two variants segregated perfectly with the dilution phenotype in their various populations. They finally suggested a combination of both variants to influence coat color dilution. In Castor and Chinchilla breeds, the c.111-5C>A variant and the c.585delG variant were in total linkage disequilibrium in diluted animals since all diluted rabbits of both breeds were homozygous mut/mut at both variant positions. The identification of other individuals mut/mut at the c.585delG variant position but homozygous wt/wt or heterozygous at the c.111-5C>A variant position might provide new insights whether c.111-5C>A has a modifying effect in the Castor and Chinchilla breeds. However, only one dilution phenotype was observed in our study, likely rejecting the involvement of the *MLPH* gene in the degree of dilution (dark vs. light dilution). The most probable hypothesis would be that the degree of dilution might result from interactions between the 1-bp deletion at the c.585delG variant, responsible for the coat color dilution, and other coat color loci.

Knowledge of the molecular mechanisms involved in the dilution phenotype is limited. Assuming that the c.585delG variant was the causal mutation affecting the trait of interest, we followed-up by analyzing the impact of the deletion on *MLPH* transcripts. Our data suggested a production of various abnormal *MLPH* transcripts in both wt and diluted rabbits. Three distinct isoforms of *MLPH* were identified in skin samples of animals: the wild type *MLPH* transcript, a shorter *MLPH* transcript corresponding to the two-exon skipping isoform and a longer *MLPH* transcript. Sequencing of this latest isoform identified a transcript with retention of intron 3 between exons 3 and 4. However, no variant was detected in the splice donor splicing or splice acceptor splicing site, which might potentially explain the retention. In both human and mouse species, various retained intron *MLPH* isoforms (ENST00000477457.1, ENST00000495439.5, ENSMUST00000136220.1) do exist, involving introns much longer than the 92-bp of intron 3 of rabbit. Our results are thus concordant with what was observed in other mammals. The qualitative presence of the exon skipping and retained intron isoforms did not seem to differ between wt and diluted rabbits. Nevertheless, the quantity seemed variable with a stronger amplification of both shorter and longer *MLPH* isoforms in individuals with a dilution of their coat color and homozygous mut/mut at both c.111-5C>A and c.585delG variants. Our data did not allow us to conclude about the direct or additional effect of the two-exon skipping on the coat color dilution or c.111-5C>A as the putative causal variant for this exon skipping since we did not investigate individuals, which were wt/wt for the c.111-5C>A variant. An in-depth analysis performed on more animals representative of the nine groups of genotypes at both variants would help to better appreciate the relative quantification of each isoform.

Finally, we showed that *MLPH* transcripts carrying the deletion were undetectable in rabbits heterozygous at the c.585delG variant while this isoform was detectable in homozygous mut/mut individuals. As expected, this result strongly suggested that the deleted *MLPH* isoform, containing a premature stop codon, went through the nonsense mediated mRNA decay (NMD) process and might be degraded. This was shown, in a proteomics experiment, for a human *MLPH* isoform (accession numbers ENST00000432475.5 and H7C052). A way to test this hypothesis would be to measure the quantity of key factors of this molecular mechanism such as up-frameshift (UPF) members [25]. In consequence, we demonstrated that the overall expression of *MLPH* transcripts, including the different isoforms, was drastically decreased in animals carrying the 1-bp deletion at the c.585delG variant and with a coat color dilution. Our results are in concordance with those obtained for other species including cats [9] and dogs [11]. Variants located within the *MLPH* gene and associated with a dilution phenotype were predicted to alter the splicing or the protein. In dogs, quantitative PCR showed that diluted animals had only about 25% of the *MLPH* transcript compared with wild type animals [11].

Together, our results bring new insights into the dilution of coat color in rabbits. The c.585delG variant remains as the most likely causative variant for coat color dilution in multiple rabbit breeds. This frameshift deletion may drive the produced *MLPH* transcript to degradation and prevent a full-length functional MLPH protein. Additional *MLPH* variants including the c.111-5C>A variant may also lead to abnormal *MLPH* isoforms (containing premature stop codon) such as exon skipping and intron retention transcripts. The production of a non-functional MLPH protein from the various *MLPH* isoforms might contribute to modifying effects on coat color by affecting melanosome transport within pigmented tissues.

## Figures and Tables

**Table 1:** Association of both variants in rabbits from Castor and Chinchilla breeds. A total of 74 individuals were genotyped including 50 Castor (37 wt vs. 13 diluted) and 24 Chinchilla (12 wt vs. 12 diluted).

		c.585delG		
		wt/wt	wt/mut	mut/mut
**c.111-5C>A**	**wt/wt**	4 Castor wt		
	
	**wt/mut**	12 Castor wt	12 Castor wt	
		2 Chinchilla wt		
	**mut/mut**	5 Castor wt	4 Castor wt	13 Castor diluted
		10 Chinchilla wt		12 Chinchilla diluted

## Supplementary Materials

The following are available online at http://www.mdpi.com/2073-4425/9/9/430/s1, Table S1: Animals used for the different experiments, Table S2: List of primers, Table S3: Haplotypes determined in Castor and Chinchilla breeds, Figure S1: cDNA sequence of the *MLPH* gene, Figure S2: Wild type MLPH protein in rabbit.
