# Supplementary material for: New Insights into the Melanophilin (MLPH) Gene Affecting Coat Color Dilution in Rabbits
Source: Genes (Basel). 2018 Aug 23;9(9):430. doi: 10.3390/genes9090430 (PMC6162760; doi:10.3390/genes9090430)
Supplement: Supplementary file 1 [file genes-09-00430-s001.pdf]

Table S1 : Animals used for the different experiments

Animal	Sexe	Breed	Phenotype	c.111-5C>A	c.585delG	Genetic analysis	Sequence analysis	MLPH transcripts analysis
1	M	Castor	diluted	mut/mut	mut/mut	x		
2	M	Castor	diluted	mut/mut	mut/mut	x		
3	F	Castor	diluted	mut/mut	mut/mut	x		
4	M	Castor	diluted	mut/mut	mut/mut	x		
5	M	Castor	diluted	mut/mut	mut/mut	x		
6	F	Castor	diluted	mut/mut	mut/mut	x		x
7	F	Castor	diluted	mut/mut	mut/mut	x	x	x
8	F	Castor	diluted	mut/mut	mut/mut	x		x
9	F	Castor	diluted	mut/mut	mut/mut	x		x
10	F	Castor	diluted	mut/mut	mut/mut	x		x
11	F	Castor	diluted	mut/mut	mut/mut	x		x
12	M	Castor	diluted	mut/mut	mut/mut	x		x
13	M	Castor	diluted	mut/mut	mut/mut	x		x
14	M	Chinchilla	diluted	mut/mut	mut/mut	x		
15	F	Chinchilla	diluted	mut/mut	mut/mut	x		
16	F	Chinchilla	diluted	mut/mut	mut/mut	x		
17	M	Chinchilla	diluted	mut/mut	mut/mut	x		x
18	M	Chinchilla	diluted	mut/mut	mut/mut	x		x
19	F	Chinchilla	diluted	mut/mut	mut/mut	x		x
20	F	Chinchilla	diluted	mut/mut	mut/mut	x	x	x
21	M	Chinchilla	diluted	mut/mut	mut/mut	x		x
22	F	Chinchilla	diluted	mut/mut	mut/mut	x		x
23	F	Chinchilla	diluted	mut/mut	mut/mut	x		x
24	F	Chinchilla	diluted	mut/mut	mut/mut	x		x
25	F	Chinchilla	diluted	mut/mut	mut/mut	x		x

26	M	Castor	wt	mut/mut	wt/mut	x		x
27	F	Castor	wt	mut/mut	wt/mut	x		x
28	F	Castor	wt	mut/mut	wt/mut	x		x
29	F	Castor	wt	mut/mut	wt/mut	x		
30	M	Castor	wt	wt/mut	wt/mut	x	x	x
31	F	Castor	wt	wt/mut	wt/mut	x		
32	F	Castor	wt	wt/mut	wt/mut	x		x
33	F	Castor	wt	wt/mut	wt/mut	x		
34	M	Castor	wt	wt/mut	wt/mut	x		x
35	M	Castor	wt	wt/mut	wt/mut	x		x
36	M	Castor	wt	wt/mut	wt/mut	x		
37	F	Castor	wt	wt/mut	wt/mut	x		
38	M	Castor	wt	wt/mut	wt/mut	x		x
39	M	Castor	wt	wt/mut	wt/mut	x		
40	F	Castor	wt	wt/mut	wt/mut	x		
41	F	Castor	wt	wt/mut	wt/mut	x		
42	F	Castor	wt	mut/mut	wt/wt	x		
43	F	Castor	wt	mut/mut	wt/wt	x		x
44	F	Castor	wt	mut/mut	wt/wt	x		x
45	F	Castor	wt	mut/mut	wt/wt	x		x
46	F	Castor	wt	mut/mut	wt/wt	x		
47	M	Castor	wt	wt/mut	wt/wt	x		x
48	M	Castor	wt	wt/mut	wt/wt	x		x
49	F	Castor	wt	wt/mut	wt/wt	x		
50	M	Castor	wt	wt/mut	wt/wt	x		x
51	F	Castor	wt	wt/mut	wt/wt	x	x	x
52	F	Castor	wt	wt/mut	wt/wt	x		
53	F	Castor	wt	wt/mut	wt/wt	x		
54	F	Castor	wt	wt/mut	wt/wt	x		
55	M	Castor	wt	wt/mut	wt/wt	x		
56	M	Castor	wt	wt/mut	wt/wt	x		x
57	F	Castor	wt	wt/mut	wt/wt	x		
58	F	Castor	wt	wt/mut	wt/wt	x		
59	M	Castor	wt	wt/wt	wt/wt	x		
60	F	Castor	wt	wt/wt	wt/wt	x		
61	M	Castor	wt	wt/wt	wt/wt	x		x
62	F	Castor	wt	wt/wt	wt/wt	x		

63	F	Chinchilla	wt	mut/mut	wt/wt	x		x
64	F	Chinchilla	wt	mut/mut	wt/wt	x		x
65	M	Chinchilla	wt	mut/mut	wt/wt	x		x
66	M	Chinchilla	wt	mut/mut	wt/wt	x		
67	F	Chinchilla	wt	mut/mut	wt/wt	x		x
68	F	Chinchilla	wt	mut/mut	wt/wt	x		x
69	M	Chinchilla	wt	mut/mut	wt/wt	x		x
70	F	Chinchilla	wt	mut/mut	wt/wt	x		
71	F	Chinchilla	wt	mut/mut	wt/wt	x		
72	F	Chinchilla	wt	mut/mut	wt/wt	x	x	x
73	F	Chinchilla	wt	wt/mut	wt/wt	x		x
74	F	Chinchilla	wt	wt/mut	wt/wt	x		x

Table S2 : List of primers

Primers have been named based on their genomic positions on the the genome assembly OryCun 2.0

Primer foward	Primer reverse	Sequence primer foward	Sequence primer reverse	PCR size	(Fontanesi et al.)	Use
549835_WT_F	550013_R	ATCCACGACCTGGACTTGG	ACTCTGCACCCTGGTCTGAA	179		gDNA genotyping, c.585delG variant
549835_DEL_F	550013_R	ATCCACGACCTGGACTTGA	ACTCTGCACCCTGGTCTGAA	179		gDNA genotyping, c.585delG variant
537310_F	537790_R	TGGACACAGTGGGAATTCAA	AAGGCGGGGCTACAAGTC	481	Ex1_1	Sequencing gDNA ex2
537446_F	537844_R	CTGCCTCTTCCTCTGTGAGC	AAAAACTCCCATGCACTGATCT	399	Ex1_2	Sequencing gDNA ex2
537168_F	537947_R	AAGCAGAGGAGGATCTGTGC	TGGCATTTCACTCTTTGACG	780		Sequencing gDNA
543667_F	544025_R	GTGAGCAGGCATGTGCAG	GGGCCTGAGCAGAGACAC	359	Ex2_1	Sequencing gDNA ex3
543872_F	544270_R	GTCAACAGCAGAAGGCAGTG	ATGCACTCACACCACCACAC	399	Ex2-3_1	Sequencing gDNA ex3-ex4
544228_F	544612_R	ACCCTGCGGGCTCCTCTA	TCTCTCTCTAGCTCTGCCTTTCA	385	Ex2-3_2	Sequencing gDNA ex3-ex4
548590_F	549052_R	GCTGGCTGGATAGATGGATG	TTGAGGTCTCCAATGGTGGT	463	Ex4_1	Sequencing gDNA ex5
548230_F	546319_R	CCCTCCCATGCTCTCTCTCT	CACCTTCTACCTGCCTCCAC	449	Ex4_2	Sequencing gDNA int4
548436_F	549032_R	GGATGAGTGGATAGGTAGGTG	AGCTCTGTTCTGTCTGGGGC	485	Ex4_3	Sequencing gDNA ex5
549663_F	550160_R	CTCCCTCAGTGCCACCTCT	TGGTCCCTAACTCCCACTTG	498	Ex5_1	Sequencing gDNA ex6
549866_F	553150_R	ATCACTCCACGTGGTCC	GACACCTCTGACGAAGGAGG	3284		Sequencing gDNA
552878_F	553424_R	TCGGGCTCTGTTTTAGCTTC	TGCAGAGCCACCACACTAAG	546		Sequencing gDNA
552946_F	553424_R	CCCCTCTCACTGTGACACC	TGCAGAGCCACCACACTAAG	479	Ex8_1	Sequencing gDNA ex9
557724_F	558123_R	AACCTTCCAGGTCAGAAACG	TGGGTGCAGATGACCTCAG	400	Ex8_2	Sequencing gDNA int9
560065_F	560521_R	GACACGTGGGACACAGAAAG	ACCTGGGAAAGAGAGGGACA	458	Ex9_1	Sequencing gDNA ex10-ex11
560343_F	560831_R	GCACAGGTACTGCCACTAAGC	CAGTGCAGCAGTGTGGAGAC	489	Ex10_1	Sequencing gDNA ex10-ex11
561834_F	562231_R	ACAGGACTCCTCCGCTCCT	GGGCAGAACTCAGGAACA	397	Ex11_1	Sequencing gDNA ex12
563173_F	563633_R	TGCACCTGAGACCCAGAGTT	ATCCCAGTCTGCCTGATGTC	461	Ex12_1	Sequencing gDNA ex13
563840_F	564225_R	GTCTCTGGGTCTGCAAGGAC	AACAACAGCAGTCGCCATC	386	Ex13_1	Sequencing gDNA ex14
563840_F	568286_R	GTCTCTGGGTCTGCAAGGAC	CTTTGGCTCGGCTGATTCT	4436	_	Sequencing gDNA
566434_F	566832_R	CCAGGTCCCCTTTAATGCT	CTTCAGGGAGAGCAAGACCA	397	Ex14_1	Sequencing gDNA ex15
567904_F	568286_R	ACCTGTGTGTCTGGGTCTCC	CTTTGGCTCGGCTGATTCT	383	Ex15_1	Sequencing gDNA ex16
568244_F	568643_R	TACCATGCGAGCCCCTCT	CACGGTACTGCGTGTCAGG	400	Ex15_2	Sequencing gDNA ex16 3'UTR
567904_F	568643_R	ACCTGTGTGTCTGGGTCTCC	CACGGTACTGCGTGTCAGG	739		Sequencing gDNA ex16 3'UTR
531653_F	549904_R	TGACTCGCGCTCTGACTG	ACAAGTCCAGGAGCTGACC	740		Sequencing cDNA ex1-ex6
544084_F	560671_R	ATGGGCTCTCTCGAGTGGTA	CTCTCTGCCTGCTGAACCTC	992		Sequencing cDNA ex4-ex11
553195_F	568072_R	GCTGGACCTCGTCTGACAGT	TCAGGGCTGCTGGGCCAT	701		Sequencing cDNA ex 9-ex16
548773_F	549904_R	GCCTTGAGGAGAGAAGTGGA	ACAAGTCCAGGAGCTGACC			cDNA genotyping, c.585delG variant
537546_F	544088_Rex3ex4	CTGGACCTTTCCAAGCTCAC	CCCATCTTCACCACTCTGGCC	335		cDNA wt MLPH isoform
531710_F	548773_Rex2ex5	GCGACCTCCACAGGGAGACCCG	CTGGCTCAGGCCCACTGCAG	171		cDNA exon-skipping MLPH isoform
543867_F	544103_R	TCCTGGTCAACAGCAGAA	TACCACTCGAGAGAGCCCAT	145		cDNA retained intron 3 MLPH isoform
HPRT_F	HPRT_R	GGCAAAACAATGCAGACCTT	CTTCGAGGGGTCCTTTTCAC	95		qPCR
544084_F	548783_R	ATGGGCTCTCTCGAGTGGTA	GGTCGCTGTCTCCACTTCTC	148		qPCR_MLPH_ex4_ex5
548773_F	549826_R	GCCTTGAGGAGAGAAGTGGA	AGGTCGTGGATGGAGAGGAG	118		qPCR_MLPH_ex5_ex6
563431_F	566531_R	TACAGAGCCTCCTGGTGAAG	ACTTCCGGTCAAGAGAGACC	107		qPCR_MLPH_ex13_ex15

<u>Table S3</u> : Haplotypes determined in Castor and Chinchilla breeds

Genomic positions correspond to positions on the the genome assembly OryCun 2.0. Amino acid positions have been determined from NP_001284414

					Chinchilla Diluted d/d		Chinchilla Wild Type D/D		Castor Diluted d/d		Castor Wild Type D/D		Castor Wild Type D/d	
**Position in OryCun 2.0**	**Type of polymorphism**	**Gene region**	**Aminoacid substitution**	**Haplotype in OryCun2.0**	**Haplotype d1**	**Haplotype d1**	**Haplotype D1**	**Haplotype D1**	**Haplotype d2**	**Haplotype d2**	**Haplotype D2**	**Haplotype D1**	**Haplotype D2**	**Haplotype d2**
537253	Y	intron1		C	T	T	T	T	T	T	C	T	C	T
537285	Y	intron1		T	C	C	C	C	C	C	T	C	T	C
537316	Y	intron1		C	T	T	T	T	T	T	C	T	C	T
537374	Y	intron1		C	T	T	T	T	T	T	C	T	C	T
537396	R	intron1		G	A	A	A	A	A	A	G	A	G	A
537421	K	intron1		G	T	T	T	T	T	T	G	T	G	T
537466	R	intron1		G	A	A	A	A	A	A	G	A	G	A
537470	Y	intron1		C	T	T	T	T	T	T	C	T	C	T
537471	R	intron1		A	G	G	G	G	G	G	A	G	A	G
537490	Y	intron1		C	T	T	T	T	T	T	C	T	C	T
537524	R	5'UTR exon2 (-10)		A	G	G	G	G	G	G	A	G	A	G
537533	R	5'UTR exon2 (-1)		G	A	A	A	A	A	A	G	A	G	A
537581	R	exon2	R16 (synonymous)	A	G	G	G	G	G	G	A	G	A	G
537660	Y	intron2		T	C	C	C	C	C	C	T	C	T	C
537673	W	intron2		A	T	T	T	T	T	T	A	T	A	T
537723	R	intron2		G	A	A	A	A	A	A	G	A	G	A
537842	Y	intron2		T	C	C	C	C	C	C	T	C	T	C
537902	Y	intron2		C	T	T	T	T	T	T	C	T	C	T
543686	complexe	intron2		359 pb	387 pb	387 pb	387 pb	387 pb	387 pb	387 pb	359 pb	387 pb	359 pb	387 pb
543688	S	intron2		C	G	G	G	G	G	G	C	G	C	G
543694	W	intron2		A	T	T	T	T	T	T	A	T	A	T
543694	W	intron2		A	T	T	T	T	T	T	A	T	A	T
543705	R	intron2		G	A	A	A	A	A	A	G	A	G	A
543705	R	intron2		G	A	A	A	A	A	A	G	A	G	A
543755	c.111-5C>A	intron2 (-5)		C	A	A	A	A	A	A	C	A	C	A
543864	R	exon3	Q72R	A	G	G	G	G	G	G	A	G	A	G
543911	R	exon3	T88A	A	G	G	G	G	G	G	A	G	A	G
544059	insG	intron3		_	insG	insG	insG	insG	insG	insG	_	insG	_	insG
544060	insC	intron3		_	insC	insC	insC	insC	insC	insC	_	insC	_	insC
544107	Y	exon4	Y122 (synonymous)	C	T	T	T	T	T	T	C	T	C	T
544110	R	exon4	E123 (synonymous)	A	G	G	G	G	G	G	A	G	A	G
544276	delT	intron4		T	delT	delT	delT	delT	delT	delT	T	delT	T	delT
544277	delC	intron4		C	delC	delC	delC	delC	delC	delC	C	delC	C	delC
544296	R	intron4		A	G	G	G	G	G	G	A	G	A	G
544579	delATTTATTT	intron4		ATTTATTT	delATTTATTT	delATTTATTT	delATTTATTT	delATTTATTT	delATTTATTT	delATTTATTT	ATTTATTT	delATTTATTT	ATTTATTT	delATTTATTT
549853	***c.585delG***	***exon6***	***frameshift***	***G***	***delG***	***delG***	***G***	***G***	***delG***	***delG***	***G***	***G***	***G***	***delG***
549877	Y	exon6	W204R	T	C	C	C	C	C	C	T	C	T	C
549909	S	exon6	S214 (synonymous)	C	G	G	G	G	G	G	C	G	C	G
550067	R	intron6		A	G	G	G	G	G	G	A	G	A	G
550127	R	intron6		G	A	A	A	A	A	A	G	A	G	A
_	R	exon7	T240A	_	G	G	G	G	G	G	A	G	A	G

_	W	exon7	P249 (synonymous)	_	T	T	T	T	T	T	A	T	A	T
_	Y	exon7	P250L	_	T	T	T	T	T	T	C	T	C	T
552962	R	intron8		A	G	G	G	G	G	G	A	G	A	G
553100	R	exon8	G298 (synonymous)	A	G	G	G	G	G	G	A	G	A	G
553111	Y	exon8	L302P	T	C	C	C	C	C	C	T	C	T	C
553147	S	exon8	G314A	G	C	C	C	C	C	C	G	C	G	C
553159	Y	exon8	V318A	T	C	C	C	C	C	C	T	C	T	C
553329	R	intron9		G	A	A	A	A	A	A	G	A	G	A
553373	R	intron9		A	G	G	G	G	G	G	A	G	A	G
553383	R	intron9		G	A	A	A	A	A	A	G	A	G	A
557796	Y	intron9		C	T	T	T	T	C	C	C	T	C	C
557804	Y	intron9		C	T	T	T	T	C	C	C	T	C	C
557823	M	intron9		C	A	A	A	A	C	C	C	A	C	C
557825	K	intron9		T	G	G	G	G	T	T	T	G	T	T
557836	S	intron9		C	G	G	G	G	C	C	C	G	C	C
557907	M	intron9		A	C	C	C	C	A	A	A	C	A	A
560156	R	intron9		G	A	A	A	A	G	G	G	A	G	G
560195	R	exon9	P343 (synonymous)	A	G	G	G	G	A	A	A	G	A	A
560207	R	exon9	A347 (synonymous)	G	A	A	A	A	G	G	G	A	G	G
560233	R	exon9	K356R	A	G	G	G	G	A	A	A	G	A	A
560258	Y	exon9	N364 (synonymous)	C	T	T	T	T	C	C	C	T	C	C
560309	Y	exon9	A381 (synonymous)	T	C	C	C	C	T	T	T	C	T	T
560534	Y	exon10	A399 (synonymous)	T	C	C	C	C	T	T	T	C	T	T
560535	R	exon10	S400G	A	G	G	G	G	A	A	A	G	A	A
560543	R	exon10	T402 (synonymous)	A	G	G	G	G	A	A	A	G	A	A
560546	Y	exon10	P403 (synonymous)	T	C	C	C	C	T	T	T	C	T	T
560621	R	exon10	A428 (synonymous)	A	G	G	G	G	A	A	A	G	A	A
560809	Y	intron11		C	T	T	T	T	C	C	C	T	C	C
563205	R	intron12		A	G	G	G	G	A	A	A	G	A	A
563248	R	intron12		A	G	G	G	G	A	A	A	G	A	A
563283	R	intron12		A	G	G	G	G	A	A	A	G	A	A
563288	Y	intron12		C	T	T	T	T	C	C	C	T	C	C
563331	Y	intron12		C	T	T	T	T	C	C	C	T	C	C
563344	K	intron12		G	T	T	T	T	G	G	G	T	G	G
563386	S	exon12	V480 (synonymous)	C	G	G	G	G	C	C	C	G	C	C
563408	R	exon12	D488S	G	A	A	A	A	G	G	G	A	G	G
563409	R	exon12	D488S	A	G	G	G	G	A	A	A	G	A	A
563428	R	exon12	P494 (synonymous)	A	G	G	G	G	A	A	A	G	A	A
563512	R	intron13		A	G	G	G	G	A	A	A	G	A	A
563513	R	intron13		A	G	G	G	G	A	A	A	G	A	A
563521	Y	intron13		T	C	C	C	C	T	T	T	C	T	T
563546	Y	intron13		T	C	C	C	C	T	T	T	C	T	T
563559	S	intron13		G	C	C	C	C	G	G	G	C	G	G
563589	W	intron13		T	A	A	A	A	T	T	T	A	T	T
563672	K	intron13		G	T	T	T	T	G	G	G	T	G	G

563796	M	intron13		C	A	A	A	A	C	C	C	A	C	C
563913-563914	delC	intron13		_	delC	delC	delC	delC	_	_	_	delC	_	_
563915-563916	delC	intron13		_	delC	delC	delC	delC	_	_	_	delC	_	_
564024	Y	intron14		C	T	T	T	T	C	C	C	T	C	C
564144	M	intron14		C	A	A	A	A	C	C	C	A	C	C
564145	Y	intron14		C	T	T	T	T	C	C	C	T	C	C
564146	R	intron14		A	G	G	G	G	A	A	A	G	A	A
564149	M	intron14		A	C	C	C	C	A	A	A	C	A	A
566515	Y	intron14		C	T	T	T	T	C	C	C	T	C	C
566561	Y	exon14	R534 (synonymous)	C	T	T	T	T	C	C	C	T	C	C
566636-566637	insC	intron15		insC	_	_	_	_	insC	insC	insC	_	insC	insC
566636-566637	insT	intron15		insT	_	_	_	_	insT	insT	insT	_	insT	insT
566694	R	intron15		G	A	A	A	A	G	G	G	A	G	G
567948	M	intron15		A	C	C	C	C	A	A	A	C	A	A
567979	Y	intron15		C	T	T	T	T	C	C	C	T	C	C
568000	W	intron15		T	A	A	A	A	T	T	T	A	T	T
568003	S	intron15		C	G	G	G	G	C	C	C	G	C	C
568117	S	exon 15 3'UTR		G	C	C	C	C	G	G	G	C	G	G
568159	W	exon 15 3'UTR		A	T	T	T	T	A	A	A	T	A	A
568160	R	exon 15 3'UTR		G	A	A	A	A	G	G	G	A	G	G
568252	R	exon 15 3'UTR		G	A	A	A	A	G	G	G	A	G	G
568328	Y	exon 15 3'UTR		T	C	C	C	C	T	T	T	C	T	T
568385	K	exon 15 3'UTR		T	G	G	G	G	T	T	T	G	T	T
568405	R	exon 15 3'UTR		G	A	A	A	A	G	G	G	A	G	G
568443	R	exon 15 3'UTR		A	G	G	G	G	A	A	A	G	A	A
568452	Y	exon 15 3'UTR		C	T	T	T	T	C	C	C	T	C	C
568470	Y	exon 15 3'UTR		T	C	C	C	C	T	T	T	C	T	T
568512	Y	exon 15 3'UTR		C	T	T	T	T	C	C	C	T	C	C
568531	S	exon 15 3'UTR		C	G	G	G	G	C	C	C	G	C	C
568537	R	exon 15 3'UTR		A	G	G	G	G	A	A	A	G	A	A
568558	Y	exon 15 3'UTR		C	T	T	T	T	C	C	C	T	C	C

<u>Figure S1</u> : cDNA sequence of the *MLPH* gene

The full cDNA *MLPH* sequence from exon 1 to exon 16 is represented with odd and even exons respectively in purple and black. The START (ATG) and STOP (TGA) codons are in black boxes. Polymorphisms that were identified are mentioned with the IUPAC ambiguity code. Pairs of primers that were used in Figure 3 are highlighted (couple 1 in yellow, couple 2 in grey, couple 3 in blue and couple 4 in pink).

AGGGTCCGGAGCGCGCCCTCAGCCTTGCGCCGCGCGCCCTGCCTGGCCCGGATCACCCCGCCTCGCCCCAGAGCGCCCTGACTCGCGCTCTGACTGGCCGCGGTCTC
TCCCTTGCAGGGCCCGGACCGGCGCTTAGCGACCTCCACAGGGAGACCCGGTGTGACCCTGCCCRGAAGCAGARATGGGGAGAAAACTGGACCTTTCCAAGCTCA
CGGACGACGAGGCCAGRCACGTCTGGGAGGTCGTGCAGCGAGACTTCGACCTCAGGAGGAAAGAGGAAGAGAGACTGCAGGGCTTGAAGGGCCAGATTCAGAGG
GAGAGCTCCAAGAGGGAGCTGCTCGCCGACACGGCGCACCTGAACGAGACGCACTGCGCCCGCTGCCTGCGGCCCTACCRGCTCCTGGTCAACAGCAGAAGGCAG
TGCCTGCACTGCGGCCTCTTCRCCTGCAGGAGCTGCAGCCGCGCCCACCCCGAGGAGCAGGGCTGGCTCTGTGACCCCTGCCACCTGGCCAGAGTGGTGAAGATGG
GCTCTCTCGAGTGGTACTAYGARCACGTGCGGGCCCGCTTCAAGCGATTCGGCAGCGCCAAGGTGATCCGGTCCCTGTACGGGCGGCTGCAGCAGGGAGGTGGGCC
TGAGCCAGGCCTTGAGGAGAGAAGTGGAGACAGCGACCTGGCAGATGAGCATGGAGAGCCAGACGCGGGGGCCCAGGCCCCACCTCTCGGCACCAAAAAAAAGC
GGCTCCTCTCCATCCACGACCTGGACTTGGAGGCGGACTCGGATCACTCCACGYGGTCCTGTGGTCAGCTCCTGGACTTGTCCTCSGAGGCTGAGGCCACGGGCAGC
CTGCAGTCCCTCACAGGTGAGCCCCACACCTGGGAGACCACCTCCCAGGAGACGRCCGTCCTGGAGGAAGCTGACCCAGGGGCCTCCGAGTGCCAGCCCCCTGCA
GAACCWCYGGGCGGCCTCTCGCCCTCCAGACGGGAGGCCCCCGAGGAGCCCTGCCAGCCCGGAACCACCAGCAGCACGGGCCTGGGGCTTGCCGCCGCTCCAGGG
ACGGCCTCTGGCAGCAGTGGRCAGCTCCCCCYGCAGTGCCAGGCCGACGTGGACACCTCTGACGAAGSAGGCACTGGGGYAGCCTCGGCGGCTGGCCCCCACCCCA
GACGCTGGAGCTGGACCTCGTCTGACAGTCAGCCCCCAGCTGCCGGGCCRCACTCAGCGGCRGACACGGAGGAGGAGGCCCTCAGGARAAGGCTGGAGGAGCTGA
CCAGCAAYGTCAGCGACCAGGAGGAGGAAGAGGGAGAGCCGGCCGGCGGGGACCACGCYGGGGCTCTCCCCCCGCGGTCCCGGCAGGACCCAGCACAGGTGTGC
ACAGACGCYRGCCCAACRCCYGGCCGGGGACAGAGCCCTCAGGGCGCCGGGGGCCCCGCCCAGCCCAGCAGGAGCACAGACGAGCAGCTGTCGGCRCTGGAGGA
CAGGGTGGCGGCCGCGGCCTCCGAGGTTCAGCAGGCAGAGAGCGAGGTGTCCGCTATCGAGTCCAGGATTGCTGCCTTGAGGGCTGCGGGGCTCACGGTGAAGCCC
TCGGGAAAGCCCCGGAGGAAATCGAACCTCCCGATCTTTGTSCCCCGGCTTCTCGGGACCCCCRRCAAGCGTCCAGAGGACCCRGATACAGAGCCTCCTGGTGAAG
CCAAGGACGAGACCGTGCCCTACCTTCTGCGGAGAAAGTACTCCCCCAGAGGTCAAGGCAAAGATGAGGTCTCTCTTGACCGGAAGTCCCCGTACCGYGGCTCCCT
GACCCAGAGGAACCCCAGCAGGAGGAGGGGAGCGGCCAGCGCCATCTTCACGAAGCCCGTGATGGCCCAGCAGCCCTGAAGGGAGCAGGCAGAGCAGCGACCT
GGCCCCACGCCGGCTTCCCTSCCCTGGCTCTCGCTGTCTGCTCTCCGTGGCCGGCGGCCCGGWRAGAAGCATCCGCCCGTGTGGCCCCCGCCTGCCAGAGCCTCAGG
CCCCTCGCTGCCCACCAGCTTCTCCGACGACCTCCAAGGCTACCATGCRAGCCCCTCTGGGCCAAGAATCAGCCGAGCCAAAGAGACTCTGGACAGTGTAGAC

<u>Figure S2</u> : Wild type Mlph protein in rabbit

```
                 10        20        30        40        50        60        70        80        90
ATGGGGAGAAAACTGGACCTTTCCAAGCTCACGGACGACGAGGCCAGRCACGTCTGGGAGGTCGTGCAGCGAGACTTCGACCTCAGGAGGAAAGAGGAAG  100
M  G  R  K  L  D  L  S  K  L  T  D  D  E  A  R  H  V  W  E  V  V  Q  R  D  F  D  L  R  R  K  E  E  E   34
AGAGACTGCAGGGCTTGAAGGGCCAGATTCAGAGGGAGAGCTCCAAGAGGGAGCTGCTCGCCGACACGGCGCACCTGAACGAGACGCACTGCGCCCGCTG  200
  R  L  Q  G  L  K  G  Q  I  Q  R  E  S  S  K  R  E  L  L  A  D  T  A  H  L  N  E  T  H  C  A  R  C    67
CCTGCGGCCCTACCRGCTCCTGGTCAACAGCAGAAGGCAGTGCCTGCACTGCGGCCTCTTCRCCTGCAGGAGCTGCAGCCGCGCCCACCCCGAGGAGCAG  300
 L  R  P  Y  X  L  L  V  N  S  R  R  Q  C  L  H  C  G  L  F  X  C  R  S  C  S  R  A  H  P  E  E  Q    100
GGCTGGCTCTGTGACCCCTGCCACCTGGCCAGAGTGGTGAAGATGGGCTCTCTCGAGTGGTACTAYGARCACGTGCGGGCCCGCTTCAAGCGATTCGGCA  400
G  W  L  C  D  P  C  H  L  A  R  V  V  K  M  G  S  L  E  W  Y  Y  E  H  V  R  A  R  F  K  R  F  G  S   134
GCGCCAAGGTGATCCGGTCCCTGTACGGGCGGCTGCAGCAGGGAGGTGGGCCTGAGCCAGGCCTTGAGGAGAGAAGTGGAGACAGCGACCTGGCAGATGA  500
  A  K  V  I  R  S  L  Y  G  R  L  Q  Q  G  G  G  P  E  P  G  L  E  E  R  S  G  D  S  D  L  A  D  E   167
GCATGGAGAGCCAGACGCGGGGGCCCAGGCCCCACCTCTCGGCACCAAAAAAAAGCGGCTCCTCTCCATCCACGACCTGGACTTGGAGGCGGACTCGGAT  600
 H  G  E  P  D  A  G  A  Q  A  P  P  L  G  T  K  K  K  R  L  L  S  I  H  D  L  D  L  E  A  D  S  D    200
CACTCCACGYGGTCCTGTGGTCAGCTCCTGGACTTGTCCTCSGAGGCTGAGGCCACGGGCAGCCTGCAGTCCCTCACAGGTGAGCCCCACACCTGGGAGA  700
H  S  T  X  S  C  G  Q  L  L  D  L  S  S  E  A  E  A  T  G  S  L  Q  S  L  T  G  E  P  H  T  W  E  T   234
CCACCTCCCAGGAGACGRCCGTCCTGGAGGAAGCTGACCCAGGGGCCTCCGAGTGCCAGCCCCCTGCAGAACCWCYGGGCGGCCTCTCGCCCTCCAGACG  800
  T  S  Q  E  T  X  V  L  E  E  A  D  P  G  A  S  E  C  Q  P  P  A  E  P  X  G  G  L  S  P  S  R  R   267
GGAGGCCCCCGAGGAGCCCTGCCAGCCCGGAACCACCAGCAGCACGGGCCTGGGGCTTGCCGCCGCTCCAGGGACGGCCTCTGGCAGCAGTGGRCAGCTC  900
 E  A  P  E  E  P  C  Q  P  G  T  T  S  S  T  G  L  G  L  A  A  A  P  G  T  A  S  G  S  S  G  Q  L    300
CCCCYGCAGTGCCAGGCCGACGTGGACACCTCTGACGAAGSAGGCACTGGGGYAGCCTCGGCGGCTGGCCCCCACCCCAGACGCTGGAGCTGGACCTCGT  1000
P  X  Q  C  Q  A  D  V  D  T  S  D  E  X  G  T  G  X  A  S  A  A  G  P  H  P  R  R  W  S  W  T  S  S   334
CTGACAGTCAGCCCCCAGCTGCCGGGCCRCACTCAGCGGCRGACACGGAGGAGGAGGCCCTCAGGARAAGGCTGGAGGAGCTGACCAGCAAYGTCAGCGA  1100
  D  S  Q  P  P  A  A  G  P  H  S  A  A  D  T  E  E  E  A  L  R  X  R  L  E  E  L  T  S  X  V  S  D   367
CCAGGAGGAGGAAGAGGGAGAGCCGGCCGGCGGGGACCACGCYGGGGCTCTCCCCCCGCGGTCCCGGCAGGACCCAGCACAGGTGTGCACAGACGCYRGC  1200
 Q  E  E  E  E  G  E  P  A  G  G  D  H  A  G  A  L  P  P  R  S  R  Q  D  P  A  Q  V  C  T  D  A  X    400
CCAACRCCYGGCCGGGGACAGAGCCCTCAGGGCGCCGGGGGCCCCGCCCAGCCCAGCAGGAGCACAGACGAGCAGCTGTCGGCRCTGGAGGACAGGGTGG  1300
P  T  P  G  R  G  Q  S  P  Q  G  A  G  G  P  A  Q  P  S  R  S  T  D  E  Q  L  S  A  L  E  D  R  V  A   434
CGGCCGCGGCCTCCGAGGTTCAGCAGGCAGAGAGCGAGGTGTCCGCTATCGAGTCCAGGATTGCTGCCTTGAGGGCTGCGGGGCTCACGGTGAAGCCCTC  1400
  A  A  A  S  E  V  Q  Q  A  E  S  E  V  S  A  I  E  S  R  I  A  A  L  R  A  A  G  L  T  V  K  P  S   467
GGGAAAGCCCCGGAGGAAATCGAACCTCCCGATCTTTGTSCCCCGGCTTCTCGGGACCCCCRRCAAGCGTCCAGAGGACCCRGATACAGAGCCTCCTGGT  1500
 G  K  P  R  R  K  S  N  L  P  I  F  V  P  R  L  L  G  T  P  X  K  R  P  E  D  P  D  T  E  P  P  G    500
GAAGCCAAGGACGAGACCGTGCCCTACCTTCTGCGGAGAAAGTACTCCCCCAGAGGTCAAGGCAAAGATGAGGTCTCTCTTGACCGGAAGTCCCCGTACC  1600
E  A  K  D  E  T  V  P  Y  L  L  R  R  K  Y  S  P  R  G  Q  G  K  D  E  V  S  L  D  R  K  S  P  Y  R   534
GYGGCTCCCTGACCCAGAGGAACCCCAGCAGGAGGAGGGGAGCGGCCAGCGCCATCTTCACGAAGCCCGTGATGGCCCAGCAGCCCTGA            1700
  G  S  L  T  Q  R  N  P  S  R  R  R  G  A  A  S  A  I  F  T  K  P  V  M  A  Q  Q  P  *               562
```
